# Typhoid Fever among Hospitalized Febrile Children in Siem Reap, Cambodia

**DOI:** 10.1093/tropej/fmr032

**Published:** 2011-09-02

**Authors:** Lalith P. M. Wijedoru, Varun Kumar, Ngoun Chanpheaktra, Kheng Chheng, Henk L. Smits, Rob Pastoor, Tran Vu Thieu Nga, Stephen Baker, Vanaporn Wuthiekanun, Sharon J. Peacock, Hor Putchhat, Christopher M. Parry

**Affiliations:** ^1^Child and Reproductive Health Group, Liverpool School of Tropical Medicine (LSTM), Liverpool, UK; ^2^Angkor Hospital for Children (AHC), Siem Reap, Cambodia; ^3^KIT Biomedical Research, Royal Tropical Institute (KIT), Amsterdam, The Netherlands; ^4^Oxford University Clinical Research Unit (OUCRU), Ho Chi Minh City, Vietnam; ^5^Mahidol-Oxford Tropical Medicine Unit (MORU), Bangkok, Thailand; ^6^Department of Medicine, University of Cambridge, Cambridge, UK

**Keywords:** typhoid fever, nucleic acid amplification test, rapid diagnostic test, blood culture, children

## Abstract

Typhoid fever was confirmed by positive blood culture in 5 (3.7%) of 134 febrile children hospitalized in Cambodia. Typhoid was suspected in an additional 25 (18.7 %) blood culture-negative children based on: a positive immunoglobulin M lateral flow assay (IgMFA) (16); a positive polymerase chain reaction (PCR) for *Salmonella typhi* (2); or clinical assessment (7). The specificity of the IgMFA and PCR assays requires further study.

## Introduction

Typhoid fever, caused by *Salmonella enterica* serovar *typhi*, is common in developing countries particularly in children [1, 2]. The current diagnostic tests for typhoid have limitations [3, 4]. In a study to assess the importance of typhoid fever in febrile children in Cambodia, we have used two novel tests in addition to blood culture.

## Methods

Cambodian children aged <16 years admitted to Angkor Hospital for Children (AHC), Siem Reap, with a documented fever of at least 38°C were eligible for enrolment. Ethical approval was obtained from the Institutional Review Board of AHC and the Research Ethics Committee of the Liverpool School of Tropical Medicine (LSTM). Informed written consent was obtained from the parents/guardians of the children.

A minimum of 1 ml venous blood (increasing with patient age) taken at admission was inoculated in brain–heart infusion broth with sodium polyanatholesulphonate and incubated at 37°C for 10 days. Bacterial isolates were identified as previously described [5].

Blood was collected on admission and on day 10 of fever or discharge, whichever was earlier, for testing with an immunoglobulin M immunochromatographic lateral flow assay (IgMFA) that detects *S. typhi* lipopolysaccharide-specific immunoglobulin M (Royal Tropical Institute) [6]. Serum (5 µl) was mixed with buffer solution (130 µl) and deposited into the IgMFA well and left for 15 min. Results were read independently by two study members blind to the clinical features. The result was scored using a scale ranging from negative, 1+ through to 4+ based on the intensity of a line present at the ‘test’ zone of the cassette compared with a reference chart produced by the manufacturer [6].

One millilitre of whole blood in ethylenediaminetetraacetic acid per patient taken at admission was used for an in-house real-time PCR assay [7]. The blood was centrifuged at 1100 relative centrifugal force for 10 minutes and the plasma and whole blood cell pellets were separated and stored at −20°C until transported frozen to OUCRU, Vietnam. DNA extraction from the cell pellet was performed under sterile conditions using the QIAamp DNA Blood Midi Kit (Qiagen). DNA was re-suspended in 300 μl of elution buffer, stored at 4°C and subjected to PCR within 24 h of preparation. The PCR for *S. typhi* and *S. paratyphi* A was performed as previously described with a calculated detection limit between 1 to 5 target copies per reaction [7].

At discharge, children were given a diagnosis of typhoid based on the results of the blood culture or the assessment of an experienced paediatrician. The diagnoses were reviewed with the result of PCR for *S. typhi* and the IgMFA.

## Results

Between April and June 2009, 148 patients were enrolled. Fourteen patients had incomplete test results due to discharge against medical advice, or study withdrawal, leaving 134 patients. The male to female ratio was 2:1 and median age was 3 years [interquartile range (IQR) 1.45–7.25]. The median days of fever when drawing IgMFA samples were 4 days (IQR 3–6.5) and 10 days (6.75–10) for admission and convalescent/discharge samples, respectively.

Fourteen (13.4%) children were considered to have clinical typhoid (Table 1). *S. typhi* was isolated from blood in five. All five had strongly positive IgMFA results (≥3+) on admission and discharge samples, with three of these demonstrating a rise in IgM titre. One child also had a positive PCR result. Two culture-negative children had a strongly positive IgMFA result (≥3+) on admission and discharge samples.

**Table 1:** Laboratory results for the 30 children with confirmed or suspected typhoid out of 134 unselected febrile children admitted to hospital in Cambodia

Patient ID	Blood culture *S. typhi*	Day of fever^a^	IgMFA^a^	Day of fever^b^	IgMFA^b^	Real-time PCR	Clinical diagnosis
1	+	8	4 +	10	4 +	−	Typhoid
2	+	6	3 +	10	4 +	−	Typhoid
3	+	7	3 +	10	3 +	+	Typhoid
4	+	6	2 +	10	3 +	−	Typhoid
5	+	3	2 +	10	3 +	−	Typhoid
6	–	3	−	6	−	+	Gastroenteritis
7	−	3	−	5	−	+	Bronchiolitis
8	−	12	4 +	14	4 +	−	Dysentery
9	−	10	3 +	12	3 +	−	Typhoid
10	−	6	3 +	10	3 +	−	Chronic arthritis
11	−	7	3 +	10	3 +	−	Typhoid
12	−	2	1 +	5	2 +	−	Gastroenteritis
13	−	3	1 +	6	2 +	−	Viral wheeze
14	−	5	1 +	10	2 +	−	Gastroenteritis
15	−	5	1 +	7	2 +	−	Viral illness
16	−	4	1 +	8	2 +	−	Gastroenteritis
17	−	4	1 +	7	1 +	−	Viral illness
18	−	2	1 +	5	1 +	−	Gastroenteritis
19	−	4	1 +	7	1 +	−	Bronchiolitis
20	−	7	−	10	1 +	−	Dengue
21	−	1	−	6	1 +	−	Gastroenteritis
22	−	7	−	10	1 +	−	Dengue
23	−	6	−	8	1 +	−	Viral illness
24	−	4	−	10	−	−	Dengue/typhoid
25	−	5	−	8	−	−	Typhoid
26	−	6	−	9	−	−	Typhoid/dengue
27	−	8	−	10	−	−	Typhoid
28	−	3	−	10	−	−	Typhoid/dengue
29	−	5	−	7	−	−	Typhoid
30	−	4	−	6	−	−	Typhoid/dengue

^a^At the time of admission to hospital.

^b^On day 10 of fever or day of discharge, whichever was earlier.

Among the 120 children with a clinical diagnosis other than typhoid, 2 had a positive PCR result but negative blood culture and IgMFA; 11 had a strongly positive IgMFA result (≥3+) on admission and discharge samples or a rise in IgM titre; and 3 had a weakly positive IgMFA result (1+) on admission and discharge samples.

## Discussion

This study suggest a lower limit on the proportion of typhoid among febrile children hospitalized in Siem Reap Cambodia of 3.7% (5/134) [8]. However, the proportion could be as much as 22.4% (30/134) if based on all three laboratory tests and clinical assessment. Blood culture is reported to be confirmatory in only 40–80% of cases [1, 3]. Sensitivity is reduced by low blood bacterial counts, low blood volumes taken for culture and pre-treatment with antimicrobials [9]. In this study, 20% of blood culture-positive children had a history of prior antimicrobial treatment compared to 33% in culture-negative clinically suspected typhoid cases. Bone marrow culture, the most sensitive method for typhoid diagnosis, is invasive and not commonly performed [10].

Reports on the sensitivity of nucleic acid amplification tests for detecting *S. typhi* in blood have been conflicting [4]. In this study, only one out of the five (20 %) blood culture-positive cases had a positive PCR, suggesting a poor sensitivity for the detection of *S. typhi* in blood. Two of the PCR-positive cases were not suspected of typhoid and were negative by IgMFA and blood culture. Contamination or the detection of dead organisms due to antimicrobial pre-treatment should be considered in these cases. The IgMFA was positive in all the blood culture-positive patients with a rising titre of IgM or strong positive result of 3+ or greater. Thirteen blood culture-negative children also had a rising IgM titre level or strong positive result of 3+ or greater, but only two of these had a clinical diagnosis of typhoid. Three further children had a weakly positive IgM at 1+ in the first and second samples. A weakly positive IgMFA result with no rise in titre may represent past but not current infection. The specificity of both assays requires further study.

Despite the diagnostic test limitations, this study suggests a significant burden of typhoid in this location that warrants further investigation.

## Funding

S.B. is funded by the OAK foundation through Oxford University. The AHC microbiology laboratory is kindly supported by the Wellcome Trust and the Li Ka Shing Foundation.
